# Breast Cancer Incidence in Sjögren Syndrome Patients

**DOI:** 10.3390/jcm14103500

**Published:** 2025-05-16

**Authors:** Jih-Jin Tsai, Li-Teh Liu, Shiow-Ing Wang, James Cheng-Chung Wei

**Affiliations:** 1 Tropical Medicine Center, Kaohsiung Medical University Hospital, No. 100, Tzyou 1st Road, Kaohsiung 80756, Taiwan; jijits@cc.kmu.edu.tw; 2 Division of Infectious Diseases, Department of Internal Medicine, Kaohsiung Medical University Hospital, No. 100, Tzyou 1st Road, Kaohsiung 80756, Taiwan; 3 School of Medicine, College of Medicine, Kaohsiung Medical University, No. 100, Shiquan 1st Road, Sanmin District, Kaohsiung 807378, Taiwan; 4 Department of Medical Laboratory Science and Biotechnology, College of Medical Technology, Chung Hwa University of Medical Technology, No. 89, Wunhua 1st Street, Rende District, Tainan 717302, Taiwan; liult0119@mail.hwai.edu.tw; 5 Center for Health Data Science, Department of Medical Research, Chung Shan Medical University Hospital, No. 110, Section 1, Jianguo N. Road, South District, Taichung 40201, Taiwan; 6 Institute of Medicine, Chung Shan Medical University, No. 110, Section 1, Jianguo N. Road, South District, Taichung 40201, Taiwan; 7 Department of Allergy, Immunology and Rheumatology, Chung Shan Medical University Hospital, No. 110, Sec. 1, Jianguo N. Road, South District, Taichung 40201, Taiwan; 8 Department of Nursing, Chung Shan Medical University, No. 110, Section 1, Jianguo N. Road, South District, Taichung 40201, Taiwan; 9 Graduate Institute of Integrated Medicine, China Medical University, No. 110, Section 1, Jianguo N. Road, South District, Taichung 40201, Taiwan

**Keywords:** breast cancer, estrogen receptor, incidence, risk factor, Sjögren syndrome

## Abstract

**Background:** The breast cancer risk profiles of SS (Sjögren syndrome) patients have shown inconsistent findings in different reports. A recent systematic review and meta-analysis indicated potential geographical variations in the link between pSS (primary Sjögren syndrome) and the risk of breast cancer. Patients with pSS from European countries exhibited a decreased likelihood of developing breast cancer, whereas an increased risk was observed in individuals from Asia and Argentina. A French study revealed that the incidence of breast cancer in pSS patients is lower. Therefore, we aimed to explore the incidence of breast cancer in SS or pSS through the TriNetX. **Methods:** Data were retrieved from 1 January 2018 to 31 December 2022. The outcome was the development of breast cancer, and Sjögren and non-Sjögren cohorts were compared. The hazard ratio (HR) and its 95% confidence interval (CI) of the outcomes were determined. A total of 5103 patients were in each cohort after propensity score matching (PSM). **Results:** We found a slightly but non-significantly elevated risk of breast cancer incidence in the Sjögren cohort (HR: 1.079, 95% CI: 0.765–1.522). The subgroup analysis showed no difference in age, race, obesity, or diabetes mellitus status. We obtained similar findings in the sensitivity analyses for pSS patients and patients in different networks. The Sjögren cohort of white patients (3.343, 1.315–8.498) and non-obese patients (4.034, 1.309–12.42) had a significantly higher risk of breast cancer occurring in overlapping sites. The risk of estrogen receptor (ER)-positive breast cancer was significantly higher among the white patients in the Sjögren cohort (1.860, 1.031–3.353). **Conclusions:** Neither SS nor pSS was significantly related to an increased risk of breast cancer, and the results according to race were similar. The white and non-obese patients in the Sjögren cohort had a significantly higher risk of breast cancer occurring in overlapping sites. White patients in the Sjögren cohort had a significantly higher risk of ER-positive breast cancer. To our knowledge, this study is the first to explore the location and ER status of breast cancer in SS patients.

## 1. Introduction

According to 2016 American College of Rheumatology (ACR) classification criteria, Sjögren syndrome (SS) encompasses primary Sjögren syndrome (pSS) [1], which develops without another underlying rheumatic condition, and secondary SS, which is linked to another rheumatic disease, such as systemic lupus erythematosus (SLE) [2], rheumatoid arthritis (RA), or systemic scleroderma (SSc). pSS is a complex autoimmune disease with multiple clinical manifestations, potentially evolving toward non-Hodgkin lymphoma with an unknown etiology. Although many potential genetic, environmental, and hormonal causes have been investigated, no causal associations currently exist that might explain the aberrant immune response to multiple epithelial structures that results in the characteristic presentation of pSS [3].

pSS is a prevalent systemic autoimmune disorder marked by dryness in exocrine glands, including the salivary and lacrimal glands, and it sometimes affects other organs beyond these glands [4,5]. pSS impacts 61 per 100,000 individuals, with a female-to-male ratio of roughly 10:1 [6]. Research has established pSS as a significant risk factor for hematological cancers [7]. A cross-sectional analysis found that breast cancer was the most prevalent solid malignancy among individuals with pSS, affecting 0.2% of patients [8]. However, female breast cancer was the most commonly diagnosed cancer, with an estimated 2.3 million new cases (11.7%) according to Global Cancer Statistics 2020 [9]. Whether or not the prevalence of breast cancer in PSS [8] was affected by the global tumor burden [9], it required further consideration. Since both pSS and breast cancer frequently affect women, understanding any potential connection between them is crucial for informing healthcare practices. However, the reported findings regarding the breast cancer profiles of SLE and pSS patients are inconsistent [10]. A recent systematic review and meta-analysis [11] indicated potential geographical variations in the link between pSS and the risk of breast cancer. Patients with pSS from European countries exhibited a decreased likelihood of developing breast cancer, whereas an increased risk was observed in individuals from Asia and Argentina.

Therefore, we aimed to explore the incidence of breast cancer in SS or pSS patients through the TriNetX platform for further investigation and clarification of this critical issue.

## 2. Materials and Methods

### 2.1. Study Design and Data Sources

This study was a retrospective cohort analysis based on aggregated data from TriNetX, a global platform that provides real-world insights within the healthcare and life sciences sectors. TriNetX includes de-identified electronic medical records (EMRs) from over 250 million individuals, sourced from more than 120 healthcare organizations worldwide. For additional details, visit their website: https://trinetx.com/?mc_cid=7e2ecd5bc5&mc_eid=%5BUNIQID%5D (accessed on 27 August 2024). The platform ensures data integrity through a standardized framework, focusing on three key quality metrics: conformance, completeness, and plausibility [12]. It has been utilized for the execution of numerous high-quality studies [13,14].

The data were collected and the analysis conducted in September 2023. For our analyses, we leveraged the U.S. Collaborative Network, a subset of the TriNetX platform, which includes data from 59 healthcare organizations. To align with our study objectives, we restricted the study period to from 1 January 2018 to 31 December 2022.

### 2.2. Study Subjects

The individuals eligible for this study were females who visited hospital at least twice during the study period. The Sjögren cohort was defined by the International Statistical Classification of Diseases, Tenth Revision, Clinical Modification (ICD-10-CM) code M35.0, as well as by a requirement for a positive Sjögren syndrome-A extractable nuclear Ab (anti-SSA/Ro) or Sjögren syndrome-B extractable nuclear Ab (anti-SSB/La; details in Supplementary Table S1) test result to establish the criteria for inclusion rigorously. For the Sjögren cohort, the index date was set as the date that the diagnosis and laboratory test results were initially fitted.

The non-Sjögren cohort was identified as individuals who received a general examination without a complaint (ICD-10-CM code Z00). They had never been diagnosed with Sjögren syndrome or had positive anti-SSA/Ro or anti-SSB/La results documented in their electronic medical records at any point in time. For the non-Sjögren cohort, the index date was established as the date of their initial encounter for the general examination.

Patients in both cohorts were excluded if they were diagnosed with breast cancer, had undergone mastectomy or breast reconstruction procedures (Supplementary Table S2), or were deceased before or on the index date. In addition, we restricted the study group to individuals aged 30 and older.

### 2.3. Ethics Approval

TriNetX complies with the Health Insurance Portability and Accountability Act (HIPAA) and the General Data Protection Regulation (GDPR). As it exclusively provides aggregated counts and statistical summaries of de-identified data, the platform has received a waiver from the Western Institutional Review Board (WIRB). Additionally, its use in this study was authorized by the Institutional Review Board of Chung Shan Medical University Hospital (CSMUH No: CS2-21176).

### 2.4. Outcomes

The primary outcome under investigation was the incidence of breast cancer (as defined by the ICD-10-CM code C50). The site at which breast cancer occurs and the specific receptors for estrogen, progesterone, or human epidermal growth factor 2 (HER2) were also explored. The patients in both cohorts were followed from one day after the index date to 5 years.

### 2.5. Covariates

To mitigate potential confounding effects, the present study included the following covariate factors, assessed within 1 year before the index date, with coding details in Supplementary Table S3.

Demographic variables included age at the index date, race (categorized as white, Black, Asian, American Indian or Alaskan Native, Native Hawaiian or Other Pacific Islander, or unknown), and socioeconomic status, and they were represented by proxy codes. The family history of primary malignant neoplasm and personal history of malignant neoplasm were also included.

The lifestyle factors analyzed included tobacco use and nicotine dependence as indicators of smoking behavior, as well as alcohol-related disorders as a proxy for alcohol consumption. To maintain a comparable health status and medical utilization across cohorts, the study also incorporated office and outpatient services, emergency department visits, hospital inpatient care, and preventive medicine services.

All comorbidities in this study were identified based on International Classification of Diseases, Tenth Revision, Clinical Modification (ICD-10-CM) codes. The conditions analyzed included neoplasms, benign breast disease, disorders of the blood and immune system, hypertensive diseases, cerebrovascular diseases, atherosclerosis, diabetes mellitus, vitamin D deficiency, overweight and obesity, hyperlipidemia, chronic lower respiratory diseases, chronic kidney diseases, menopausal and female climacteric states, infertility, noninfective enteritis and colitis, diseases of liver, sleep disorders, depression, anxiety, dissociative, stress-related, somatoform, and other nonpsychotic mental disorders, rheumatoid arthritis, systemic lupus erythematosus, and disorders of bone density and structure.

Procedures such as breast mammography, radiation treatment management, and radiation treatment delivery were also considered (Supplementary Table S2). We also considered using medications, which were determined using the Anatomical Therapeutic Chemical (ATC) code. In this study, we included the following medications for analysis: corticosteroids for systemic use, anti-inflammatory and anti-rheumatic products, non-steroidal anti-inflammatory drugs (NSAIDs), estrogens, hormonal contraceptives for systemic use, progestogens, calcium, vitamin D, and bisphosphonates. The laboratory results for Sjögren syndrome-A extractable nuclear Ab, Sjögren syndrome-B extractable nuclear Ab are also included in Table 1 and Supplementary Table S1.

### 2.6. Statistical Analyses

To mitigate the influence of confounding factors, we used a built-in tool from TriNetX to help us predict which group each person is more likely to belong to, based on basic information like age, gender, and medical history. We gave each person a “propensity score”, which is like a number showing how likely they are to fit into a certain group. Then, we matched each person with another person who was the most similar to them. Each person was matched with only one other person (1:1 matching). This method helped to make sure the two groups were as similar as possible in their background (https://support.trinetx.com/hc/en-us/articles/360011978033-In-compare-outcomes-how-are-patients-matched-when-balancing-cohorts/, accessed on 27 August 2024). A caliper of 0.1 was used to pool the standard deviations of the two groups during the matching process for variables such as age at index, race, socioeconomic status, family history of malignant neoplasm, personal history of malignant neoplasm, lifestyle, breast mammography, and medical utilization. We evaluated the similarity between the two groups before and after matching using standardized mean differences (SMDs), with an SMD below 0.1 signifying a well-balanced comparison between cohorts.

We applied Kaplan–Meier analysis to estimate outcome probabilities. At the same time, hazard ratios (HRs), confidence intervals (CIs), and proportionality tests were calculated using R’s Survival package v3.2-3 (https://support.trinetx.com/hc/en-us/articles/360053133594-How-does-TriNetX-test-for-proportionality-on-a-hazard-ratio-, accessed on 27 August 2024). The log-rank test was conducted to assess differences in survival curves between cohorts, with the results analyzed using the TriNetX platform.

Additionally, we performed five subgroup analyses to investigate variations between cohorts, including age at index (30–64 years/≥65 years), race (white/Black or African American/Asian), obesity (with/without), diabetes mellitus (DM) (with/without), and bone disorders (with/without).

Three sensitivity values were also obtained. First, to better explore the impact of Sjögren syndrome, subjects were excluded if they had other comorbid autoimmune diseases (such as rheumatoid arthritis, ankylosing spondylitis, psoriasis, systemic lupus erythematosus, dermato- or poly-myositis, systemic sclerosis, other overlap syndromes, Behçet’s disease, polyarteritis nodosa or related conditions, other necrotizing vasculopathies, vasculitis limited to the skin not elsewhere classified, noninfective enteritis and colitis, celiac disease, or type 1 diabetes mellitus). Second, we modified the definition of Sjögren syndrome by adopting a broader definition (diagnosed with ICD-10 code M35.0 at least twice). In addition, to explore geographic differences, we performed the same study design using different networks (the global network/US network/Asia–Pacific and APAC networks).

## 3. Results

### 3.1. Profile of Study Participants

**Figure 1:**
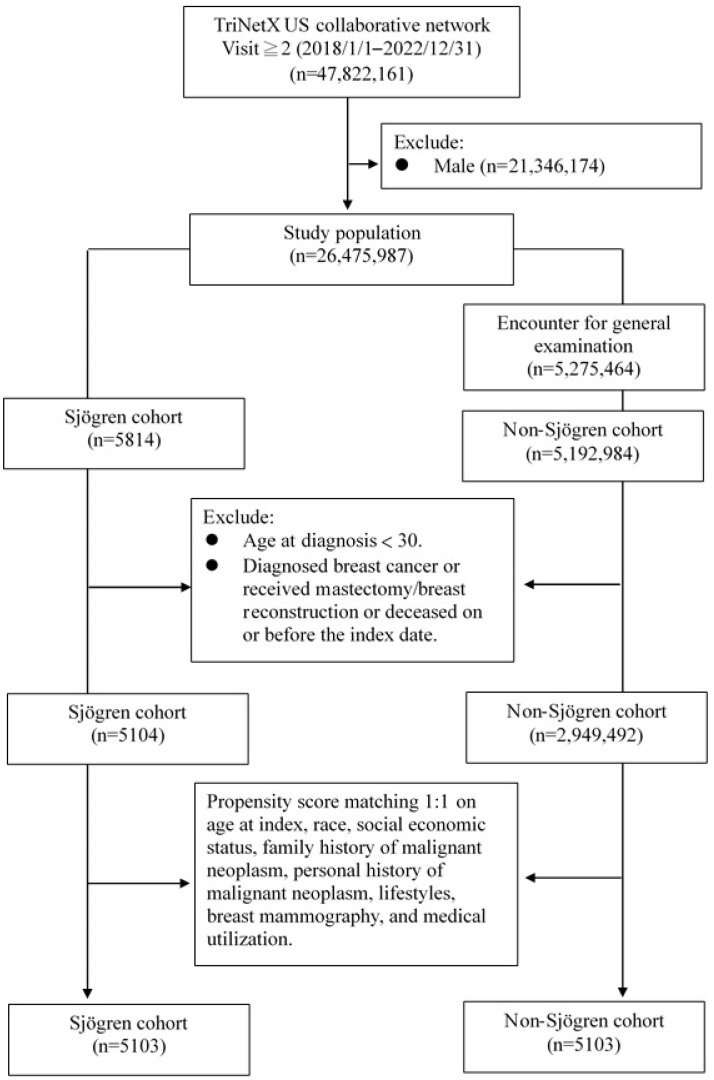
Selection process.

Following propensity score matching, 5103 patients were assigned to the Sjögren cohort, matched by an equivalent number in the non-Sjögren cohort. The selection procedure is depicted in Figure 1.

The baseline characteristics of the study participants are presented in Table 1, which shows data before and after the matching process. Propensity score matching was conducted based on age, race, socioeconomic status, family history of cancer, personal cancer history, lifestyle factors, breast mammography, and healthcare utilization. After matching, the differences were significantly reduced, with standardized mean differences (SMDs) for the matched variables below 0.1, within an acceptable range.

### 3.2. Outcomes

**Figure 2:**
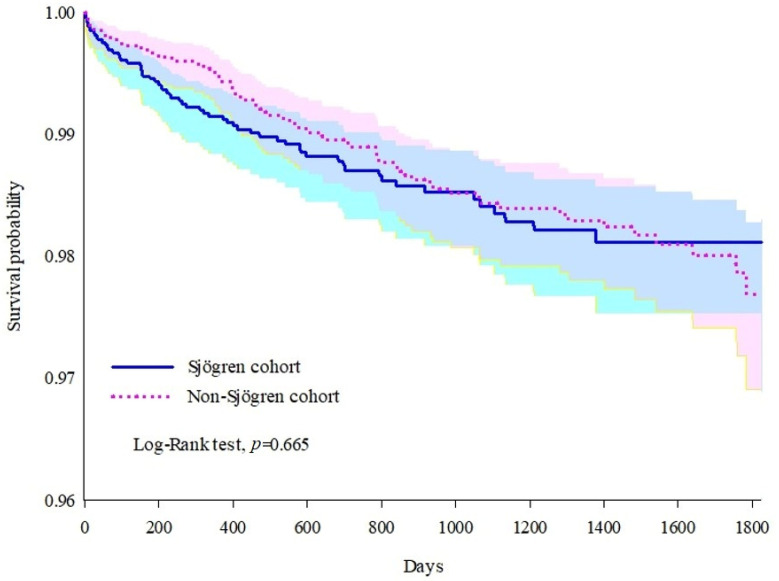
Kaplan–Meier curve for incidence of breast cancer.

Table 2 showed the count of patients with outcomes in both cohorts and the 5-year adjusted hazard ratios for the incidence of breast cancer in the Sjögren cohort relative to the non-Sjögren cohort. There was a slightly higher risk of an incidence of breast cancer in the Sjögren cohort (HR: 1.079, 95% CI: 0.765–1.522) than in the non-Sjögren cohort, but the difference was not significant, and it is also illustrated in Figure 2 by the Kaplan–Meier curve for the incidence of breast cancer.

From the perspective of the site of the occurrence of breast cancer (Table 2), the Sjögren cohort had a slightly higher risk of breast cancer in the central portion, upper inner, lower inner, lower outer quadrant, overlapping sites, and unspecified sites. Still, the difference was insignificant (HR: 2.663, 1.282, 2.910, 1.189, 1.914, and 1.256, respectively).

In addition, compared to the non-Sjögren cohort, the Sjögren cohort had a higher risk of estrogen receptor positivity in breast cancer (HR: 1.222, 95% CI: 0.782–1.907) but a lower risk of progesterone receptor positivity (HR: 0.531, 95% CI: 0.048–5.861), although neither reached statistical significance.

We consistently observed similar patterns when we accounted for multiple variables (refer to Supplementary Table S4). Compared to the non-Sjögren cohort, the Sjögren cohort had a significantly higher risk of breast cancer occurring in the upper inner quadrant (HR: 3.256, 95% CI: 1.032–10.27) and estrogen receptor positivity (HR: 1.640, 95% CI: 1.014–2.653) in Model 4, in which comorbidities were added and medicine was used in combination with the previous propensity score matching.

Similar patterns were observed for different follow-up durations (Supplementary Table S5). Compared to the non-Sjögren cohort, the Sjögren cohort had a significantly higher risk of breast cancer in terms of estrogen receptor positivity, especially at shorter follow-up times (180 days, 1 year) (HR: 3.627, 2.765, respectively).

#### 3.2.1. Subgroup Analyses

##### Age

The analysis of breast cancer risk was conducted by categorizing the study participants into age-based subgroups (Supplementary Table S6). Whether the women were aged 30–64 years or 65 years and above, the incidence of breast cancer showed no notable variation between those in the Sjögren cohort and those in the non-Sjögren cohort.

##### Race

We then proceeded with a more in-depth investigation according to race (Supplementary Table S7). There was no significant difference in the occurrence of breast cancer between the Sjögren cohort and the non-Sjögren cohort among Black, African Americans, or Asian women. However, the Sjögren cohort had a significantly higher risk of breast cancer occurring in overlapping sites (HR: 3.343, 95% CI: 1.315–8.498) and estrogen receptor positivity (HR: 1.860, 95% CI: 1.031–3.353) among the white population.

##### Obesity

Obesity is a risk factor for breast cancer, so we conducted a stratified analysis based on whether the individual was obese (Supplementary Table S8). There was no significant difference in the occurrence of breast cancer between the Sjögren cohort and the non-Sjögren cohort among obese subjects. However, the Sjögren cohort had a significantly higher risk of breast cancer occurring in overlapping sites (HR: 4.034, 95% CI: 1.309–12.42) among non-obese subjects.

##### Diabetes Mellitus (DM)

There was a slightly elevated risk of incident breast cancer in the Sjögren cohort (HR: 2.732, 95% CI: 0.704–10.59) compared to the non-Sjögren cohort, but this difference was not significant (Supplementary Table S9). There was no significant difference in the occurrence of breast cancer between the Sjögren cohort and the non-Sjögren cohort among subjects without DM.

##### Bone Density and Structure Disorders

There was no significant difference in the occurrence of breast cancer between the Sjögren cohort and the non–Sjögren cohort, regardless of whether the patients had comorbidities related to bone density or structural disorders (Supplementary Table S10).

**Figure 3:**
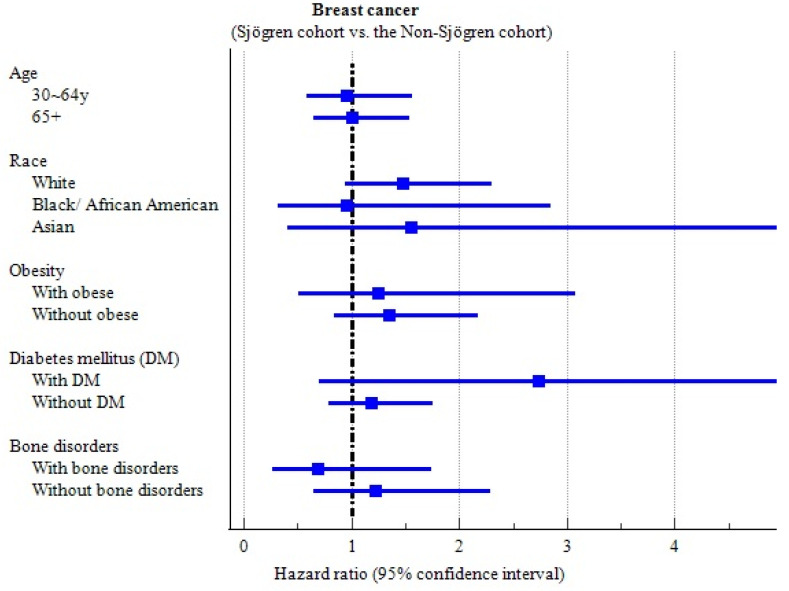
Forest plot of breast cancer’s incidence in subgroup analyses.

In Figure 3, the forest plot of breast cancer’s incidence summarizes the overall results of the subgroup analyses.

#### 3.2.2. Sensitivity Analyses

We further conducted three sensitivity analyses to test the robustness of the results. We obtained similar results when we excluded subjects with comorbidities with other autoimmune diseases (Supplementary Table S11). When we used the modified Broder definition for the Sjögren cohort, there was no significant difference in the occurrence of breast cancer between the Sjögren cohort and the non-Sjögren cohort (Supplementary Table S12), except for the occurrence at the central site (HR: 0.655, 95% CI: 0.445–0.964). A similar pattern was observed when we applied the same study design to different networks (Supplementary Table S13). Moreover, there was no significant difference in the occurrence of breast cancer between the Sjögren cohort and the non-Sjögren cohort, regardless of whether the global network, the U.S. network, or the APAC network was used.

## 4. Discussion

Our study showed that the risk of an incidence of breast cancer was slightly higher in the Sjögren cohort (HR: 1.079, 95% CI: 0.765–1.522) than in the non-Sjögren cohort but was not significantly different. A subgroup analysis of age, race (white, Black or African American, or Asian), obesity status, DM status, and bone disorders revealed no difference in the incidence of breast cancer between these two cohorts. According to the sensitivity analyses for pSS on different networks (the global, US, or APAC network), we also obtained similar findings, indicating no significant difference in the occurrence of breast cancer between the Sjögren cohort and the non-Sjögren cohort.

A recent study [15] in 2022 revealed that patients with five major autoimmune diseases (AIDs), namely, SLE, RA, SS, SSc, and idiopathic inflammatory myositis (IIM), in China had an increased risk of developing cancer, with a predominance of women and younger patients. Among the five major AIDs, IIM had the highest SIR (standardized incidence ratio) (4.31, 3.34–5.48), followed by RA (3.99, 3.40–4.65), SSc (3.77, 2.49–5.49), SS (2.88, 2.30–3.56), and SLE (2.58, 2.07–3.17). Patients with SS had significantly high SIRs for developing NHL (non-Hodgkin lymphoma) (24.88, 12.42–44.51) and solid tumors, including thyroid (8.41, 4.34–14.68), pancreas (6.86, 2.23–16.01), urinary tract (6.29, 1.30–18.39), cervical (4.18, 1.53–9.1), colon (4.06, 1.49–8.83), and lung (2.44, 1.26–4.26) cancers. An increased risk of breast cancer was found in patients with IIM (4.52, 2.07–8.59), RA (3.85, 2.15–6.35), and SSc (3.93, 1.07–10.07), but was not observed in those with SLE and SS.

The findings of various reports about breast cancer risk profiles in SLE and pSS patients have been inconsistent [10]. A recent systematic review and meta-analysis [11] suggested that there may be geographical differences in the association between pSS and the risk of breast cancer. pSS in European patients was associated with a lower risk of breast cancer (0.61, 0.51–0.73), while the opposite was shown in patients from Asia (1.32, 1.10–1.58) or Argentina (3.76, 1.04–9.45). The pooled result from 28,635 female pSS patients indicated that the incidence of breast cancer was 2.15 (95% CI: 1.33–3.50) per 1000 person/years. A French study [16] revealed that pSS patients had higher incidences of lymphoma (1.97, 1.59–2.43), Waldenström macroglobulinemia (10.8, 6.5–18.0), and leukemia (1.61, 1.1–2.4). The incidence of thyroid cancer was higher (1.7, 1.1–2.8), whereas the incidences of bladder and breast cancer were lower (0.58, 0.37–0.89 and 0.60, 0.49–0.74, respectively). From the previous studies, geographical and ethnic differences played a role in the risk of breast cancer in pSS cohorts. However, in our study, we did not find that SS or pSS was significantly related to an increased risk of breast cancer. The result was the same among the different races examined, including white (66.2%), Black or African American (15.6%), and Asian patients (4.7%).

Our Sjögren cohort constructed with Model 4 (Supplementary Table S4) had a significantly higher risk of breast cancer in the upper inner quadrant (3.256, 1.032–10.27) and estrogen receptor positivity (1.640, 1.014–2.653). Considering different follow-up durations (Supplementary Table S5), the Sjögren cohort had a significantly higher risk of breast cancer due to estrogen receptor positivity, especially at shorter follow-up times (180 days and 1 year) (HR: 3.627 and 2.765, respectively).

Compared to Black, African American, or Asian patients, white patients in the Sjögren cohort had a significantly higher risk of breast cancer in overlapping sites (3.343, 1.315–8.498) and positive results for estrogen receptors (1.860, 1.031–3.353). Additionally, the Sjögren cohort had a significantly higher risk of breast cancer occurring in overlapping sites (4.034, 1.309–12.42) among non-obese subjects.

According to our results, the Sjögren cohort of white patients (3.343, 1.315–8.498) and non-obese patients (4.034, 1.309–12.42) had a significantly higher risk of breast cancer occurring at overlapping sites. In Model 4 (Supplementary Table S4), the Sjögren cohort had a significantly higher risk of incident breast cancer in the upper inner quadrant (3.256, 1.032–10.27). The location of the primary tumor could play a crucial role in influencing the prognosis of breast cancer patients. A previous study [17] found that patients with tumors located in overlapping lesions had increased odds of positive axillary lymph nodes (1.58, 1.36–1.83) and a higher risk of mortality (1.28, 1.05–1.55). Conversely, those with tumors in the upper inner quadrant (0.68, 0.56–0.84) or lower inner quadrant (0.72, 0.56–0.93) had reduced odds of positive axillary lymph nodes.

In our study, the Sjögren cohort had a significantly higher risk of ER-positive breast cancer according to Model 4 (1.640, 1.014–2.653), white race (1.860, 1.031–3.353), and shorter follow-up times (180 days, 1 year) (3.627 and 2.765, respectively). Most breast cancers overexpress the estrogen receptor (ER) and progesterone receptor (PR). The creation of medications like tamoxifen, designed to target hormone receptors, has greatly enhanced survival rates among women diagnosed with hormone receptor-positive breast cancer [18]. The ER is the earliest breast cancer biomarker studied and is expressed in approximately 70–84% of breast cancer patients. ER-positive breast cancer, in contrast to ER-negative breast cancer, is generally more well differentiated, less aggressive, and associated with an improved prognosis and overall survival rates [19]. ER serves as the key predictive biomarker for all endocrine therapies across both early and advanced stages of ER-positive breast cancer. The use of adjuvant tamoxifen-based treatment has resulted in a 29% decrease in mortality among patients with advanced ER-positive breast cancer.

The protein tripartite motif-containing 21 (TRIM21/Ro52) plays a vital role in antigen presentation and in modulating innate immunity to combat intracellular pathogens. Additionally, it acts as a negative regulator of interferon production [10]. TRIM21/Ro52 is frequently identified as an autoantigen in various systemic autoimmune diseases, particularly in patients with SLE and pSS. Research indicates that TRIM21/Ro52 contributes to the pathology of pSS and cancer, with increased expression levels linked to improved survival outcomes in certain cancer types, including diffuse large B-cell lymphoma (DLBCL), breast cancer, and renal cell carcinoma [20,21,22]. Conversely, TRIM21/Ro52 enhances the proliferation and migration of cancer cells in glioma and thyroid cancer, while also contributing to increased drug resistance in colorectal and pancreatic cancers [23,24,25]. Further investigation is needed to uncover the mechanisms by which TRIM21/Ro52 contributes to cancer’s progression or offers protective effects against cancer in patients with SLE and pSS.

Our study has certain limitations. Firstly, as over 80% of the study participants were American and fewer than 5% were Asian, the applicability of our findings to Asian and European populations remains constrained. Secondly, despite utilizing validated outcome definitions and propensity score matching to minimize bias, the inherent limitations of studies based on electronic medical records (EMRs) make it impossible to fully eliminate misclassification bias and residual confounding. Third, TriNetX data originates from hospital-based EMRs rather than from population-based data. Fourth, in our Sjögren cohort, the index date was set as the date on which the diagnosis and laboratory test results were initially fitted. Therefore, we presumed that SS was the cause of breast cancer, and we may have missed patients diagnosed with breast cancer before the diagnosis of pSS. Specifically, a diagnosis of pSS might be ignored or delayed due to an inconspicuous presentation. Therefore, we might also ignore patients with the paraneoplastic syndrome of SS.

## 5. Conclusions

In conclusion, we did not find that SS or pSS was significantly related to an increased risk for breast cancer, and our analysis of race yielded similar results. The white patients in the Sjögren cohort or non-obese subjects had a significantly higher risk of breast cancer occurring in overlapping sites. Breast cancer located in overlapping lesions had increased odds of positive axillary lymph nodes and a higher risk of mortality. The Sjögren cohort had a significantly higher risk of breast cancer associated with ER positivity, either in the white population or at shorter follow-up times (180 days, 1 year). ER-positive breast cancer, in contrast to ER-negative breast cancer, is generally more well differentiated, less aggressive, and associated with an improved prognosis and overall survival rates. To our knowledge, this is the first study to explore the location and extent of ER-positive breast cancer in SS patients. The influence of the tumor’s location and the presentation of ER and PR in breast cancer in pSS patients needs to be further investigated. Future research is needed to further characterize the effect of pSS on the risk of breast cancer and other malignancies and explore the roles of genetic background and other risk factors among different ethnicities, TRIM/Ro52, and pathophysiological mechanisms.

## Figures and Tables

**Table 1:** Baseline characteristics of study subjects (before and after matching).

Variables	Before Matching			After Matching ^a^		
	Sjögren Cohort (n = 5104)	Non-Sjögren Cohort (n = 2,949,492)	SMD	Sjögren Cohort (n = 5103)	Non-Sjögren Cohort (n = 5103)	SMD
Age at index						
Mean ± SD	56.0 ± 14.1	54.4 ± 14.8	**0.107**	55.9 ± 14.1	56.0 ± 14.1	0.004
Race, n (%)						
White	3380 (66.2)	2,022,273 (68.6)	0.050	3380 (66.2)	3380 (66.2)	<0.001
Black or African American	795 (15.6)	430,111 (14.6)	0.028	794 (15.6)	783 (15.3)	0.006
Asian	240 (4.7)	119,417 (4.0)	0.032	240 (4.7)	244 (4.8)	0.004
American Indian or Alaska Native	25 (0.5)	8735 (0.3)	0.031	25 (0.5)	18 (0.4)	0.021
Native Hawaiian or Other pacific islander	13 (0.3)	6776 (0.2)	0.005	13 (0.3)	22 (0.4)	0.030
Unknown	651 (12.8)	362,180 (12.3)	0.014	651 (12.8)	656 (12.9)	0.003
Social economic status						
Housing/economic circumstances problem	17 (0.3)	5906 (0.2)	0.026	17 (0.3)	14 (0.3)	0.011
Problems related to education and literacy	16 (0.3)	584 (0.0)	0.072	15 (0.3)	15 (0.3)	<0.001
Cancer history						
Family history of primary malignant neoplasm	225 (4.4)	93,139 (3.2)	0.066	225 (4.4)	210 (4.1)	0.015
Personal history of malignant neoplasm	213 (4.2)	81,314 (2.8)	0.077	213 (4.2)	206 (4.0)	0.007
Lifestyles						
Nicotine dependence	254 (5.0)	113,520 (3.8)	0.055	254 (5.0)	260 (5.1)	0.005
Tobacco use	87 (1.7)	43,328 (1.5)	0.019	87 (1.7)	95 (1.9)	0.012
Alcohol-related disorders	29 (0.6)	19,260 (0.7)	0.011	29 (0.6)	25 (0.5)	0.011
Medical utilization						
Office or other outpatient services	3467 (67.9)	1,379,313 (46.8)	**0.438**	3466 (67.9)	3487 (68.3)	0.009
Emergency department services	1019 (20.0)	312,433 (10.6)	**0.263**	1018 (19.9)	1002 (19.6)	0.008
Preventive medicine services	551 (10.8)	181,861 (6.2)	**0.167**	551 (10.8)	538 (10.5)	0.008
Hospital inpatient services	381 (7.5)	100,862 (3.4)	**0.179**	381 (7.5)	377 (7.4)	0.003
Comorbidities						
Neoplasms	864 (16.9)	294,100 (10.0)	**0.205**	863 (16.9)	674 (13.2)	**0.104**
Benign neoplasm of breast	15 (0.3)	6216 (0.2)	0.017	15 (0.3)	13 (0.3)	0.007
Diseases of the blood and blood-forming organs and certain disorders involving the immune mechanism	1378 (27.0)	251,953 (8.5)	**0.498**	1377 (27.0)	638 (12.5)	**0.370**
Hypertensive diseases	1645 (32.2)	728,354 (24.7)	**0.168**	1645 (32.2)	1612 (31.6)	0.014
Cerebrovascular diseases	268 (5.3)	66,577 (2.3)	**0.158**	268 (5.3)	165 (3.2)	**0.100**
Atherosclerosis	153 (3.0)	29,529 (1.0)	**0.143**	152 (3.0)	68 (1.3)	**0.114**
Diabetes mellitus	509 (10.0)	285,693 (9.7)	0.010	509 (10.0)	640 (12.5)	0.081
Vitamin D deficiency	767 (15.0)	219,544 (7.4)	**0.242**	767 (15.0)	511 (10.0)	**0.152**
Overweight and obesity	647 (12.7)	272,708 (9.2)	**0.110**	646 (12.7)	610 (12.0)	0.021
Hyperlipidemia, unspecified	695 (13.6)	360,887 (12.2)	0.041	694 (13.6)	801 (15.7)	0.059
Female infertility	13 (0.3)	8847 (0.3)	0.009	13 (0.3)	12 (0.2)	0.004
Chronic lower respiratory diseases	812 (15.9)	257,291 (8.7)	**0.220**	811 (15.9)	584 (11.4)	**0.130**
Chronic kidney disease	331 (6.5)	90,056 (3.1)	**0.162**	331 (6.5)	198 (3.9)	**0.118**
Menopausal and female climacteric states	86 (1.7)	34,366 (1.2)	0.044	86 (1.7)	97 (1.9)	0.016
Noninfective enteritis and colitis	194 (3.8)	55,381 (1.9)	**0.116**	194 (3.8)	126 (2.5)	0.077
Diseases of liver	363 (7.1)	68,214 (2.3)	**0.228**	363 (7.1)	156 (3.1)	**0.185**
Sleep disorders	751 (14.7)	197,448 (6.7)	**0.262**	750 (14.7)	474 (9.3)	**0.167**
Depressive episode	647 (12.7)	223,185 (7.6)	**0.170**	646 (12.7)	520 (10.2)	0.078
Anxiety, stress-related, or nonpsychotic mental disorders	928 (18.2)	366,576 (12.4)	**0.160**	928 (18.2)	822 (16.1)	0.055
Rheumatoid arthritis with rheumatoid factor	243 (4.8)	12,580 (0.4)	**0.275**	242 (4.7)	27 (0.5)	**0.265**
Other rheumatoid arthritis	555 (10.9)	29,604 (1.0)	**0.427**	554 (10.9)	69 (1.4)	**0.405**
Systemic lupus erythematosus	708 (13.9)	10,468 (0.4)	**0.545**	707 (13.9)	27 (0.5)	**0.534**
Disorders of bone density and structure	762 (14.9)	188,042 (6.4)	**0.280**	761 (14.9)	422 (8.3)	**0.209**
Procedures						
Breast mammography	968 (19.0)	421,820 (14.3)	**0.125**	967 (18.9)	963 (18.9)	0.002
Radiation treatment management	10 (0.2)	2916 (0.1)	0.025	10 (0.2)	10 (0.2)	<0.001
Radiation treatment delivery	10 (0.2)	2287 (0.1)	0.032	10 (0.2)	10 (0.2)	<0.001
Medications						
Corticosteroids for systemic use	2188 (42.9)	580,727 (19.7)	**0.516**	2188 (42.9)	1329 (26.0)	**0.360**
NSAIDs	1425 (27.9)	488,717 (16.6)	**0.275**	1425 (27.9)	1136 (22.3)	**0.131**
Estrogens	331 (6.5)	144,281 (4.9)	0.069	331 (6.5)	311 (6.1)	0.016
Hormonal contraceptives for systemic use	196 (3.8)	133,331 (4.5)	0.034	196 (3.8)	274 (5.4)	0.073
Progestogens	150 (2.9)	80,356 (2.7)	0.013	150 (2.9)	162 (3.2)	0.014
Calcium	701 (13.7)	226,215 (7.7)	**0.197**	701 (13.7)	548 (10.7)	0.092
Vitamin D and analogues	612 (12.0)	161,986 (5.5)	**0.232**	612 (12.0)	399 (7.8)	**0.140**
Bisphosphonates	140 (2.7)	37,181 (1.3)	**0.106**	140 (2.7)	80 (1.6)	0.081
Laboratory						
Testosterone, ≥0.6 ng/dL	29 (0.6)	8625 (0.3)	0.042	29 (0.6)	16 (0.3)	0.038
Mean ± SD	38.36 ± 76.72	50.88 ± 109.7	0.132	38.36 ± 76.97	41.64 ± 83.60	0.040
Sjogren syndrome-A extractable nuclear Ab (arb’U/mL)						
Mean ± SD	24.15 ± 61.20	0.848 ± 1.628	0.538	24.15 ± 61.20	0.591 ± 1.162	0.544
Sjogren syndrome-B extractable nuclear Ab (arb’U/mL)						
Mean ± SD	10.96 ± 55.47	0.783 ± 1.460	0.259	10.96 ± 55.47	0.447 ± 0.987	0.268

Bold font represents a standardized difference more than 0.1. If the patients are less or equal to 10, results show the count as 10. SD: Standard deviation. SMD: standardized mean difference, NA: not available. NSAIDs: anti-inflammatory and anti-rheumatic products, non-steroids. ^a^ Propensity score matching was performed on age at index, race, social economic status, family history of malignant neoplasm, personal history of malignant neoplasm, lifestyles, breast mammography, and medical utilization.

**Table 2:** Risk of outcome (1 day to 5 years).

Outcomes	Patients with Outcome		Adjusted Hazard Ratio (95% CI) ^a^
	Sjögren Cohort (n = 5103)	Non-Sjögren Cohort (n = 5103)	
Breast cancer	63	68	1.079 (0.765–1.522)
Site			
Nipple and areola	10	10	0.806 (0.225–2.886)
Central portion	10	10	2.663 (0.793–8.943)
Upper inner quadrant	11	10	1.282 (0.543–3.029)
Lower inner quadrant	10	10	2.910 (0.562–15.06)
Upper outer quadrant	17	26	0.789 (0.427–1.457)
Lower outer quadrant	10	10	1.189 (0.382–3.702)
Axillary tail of breast	0	10	NA
Overlapping sites	21	13	1.914 (0.956–3.831)
Unspecified site	59	55	1.256 (0.869–1.816)
Receptor			
HER2-positive	0	0	NA
Estrogen receptor-positive	40	38	1.222 (0.782–1.907)
Progesterone receptor-positive	10	10	0.531 (0.048–5.861)

Note: CI: confidence interval. HER2: human epidermal growth factor receptor 2. NA: not available. If the patient number is less or equal to 10, the results show the count as 10. ^a^ Propensity score matching was performed on age at index, race, social economic status, family history of malignant neoplasm, personal history of malignant neoplasm, lifestyles, breast mammography, and medical utilization.

## Data Availability

The data supporting this study’s findings can only be obtained on a reasonable request. Further information about the data of TriNetX can be accessed on their website: https://trinetx.com/?mc_cid=7e2ecd5bc5&mc_eid=%5BUNIQID%5D (accessed on 27 August 2024).

## Supplementary Materials

The following supporting information can be downloaded at: https://www.mdpi.com/article/10.3390/jcm14103500/s1, Supplementary Table S1. Sjögren syndrome-A extractable nuclear Ab or Sjögren syndrome-B extractable nuclear Ab codes used in the present study. Supplementary Table S2. Mastectomy or breast reconstruction procedure codes. Supplementary Table S3. Covariates of characteristics of study subjects. Supplementary Table S4. Risk of outcome-adjusted variables. Supplementary Table S5. Risk of outcomes for different follow-up durations. Supplementary Table S6. Risk of outcome (1 day to 5 years) stratified by age. Supplementary Table S7. Risk of outcome (1 day to 5 years) stratified by race. Supplementary Table S8. Risk of outcome (1 day to 5 years) stratified by obesity. Supplementary Table S9. Risk of outcome (1 day to 5 years) stratified by DM. Supplementary Table S10. Risk of outcome (1 day to 5 years) stratified by bone density and structure disorders. Supplementary Table S11. Sensitivity analysis—exclusion of subjects with comorbidities related to other autoimmune diseases. Supplementary Table S12. Sensitivity analysis—modified definition of Sjögren cohort. Supplementary Table S13. Sensitivity analysis—application of same study design to different networks.
