# Supplementary material for: Breast Cancer Incidence in Sjögren Syndrome Patients
Source: J Clin Med. 2025 May 16;14(10):3500. doi: 10.3390/jcm14103500 (PMC12111816; doi:10.3390/jcm14103500)
Supplement: Supplementary file 1 [file jcm-14-03500-s001.zip › jcm-3579070-supplementary.pdf]

# Supplementary Materials

## Table of contents

Page 2: **Supplementary Table S1**. Sjögren syndrome-A extractable nuclear Ab or Sjögren syndrome-B extractable nuclear Ab codes used in the present study

Page 4: **Supplementary Table S2**. Mastectomy or breast reconstruction procedure codes

Page 5: **Supplementary Table S3**. Covariates of characteristics of study subjects

Page 5: **Supplementary Table S4**. Risk of outcome-adjusted variables

Page 6: **Supplementary Table S5**. Risk of outcomes for different follow-up durations

Page 7: **Supplementary Table S6**. Risk of outcome (1 day to 5 years) stratified by age

Page 8: **Supplementary Table S7**. Risk of outcome (1 day to 5 years) stratified by race

Page 9: **Supplementary Table S8**. Risk of outcome (1 day to 5 years) stratified by obesity

Page 10: **Supplementary Table S9**. Risk of outcome (1 day to 5 years) stratified by DM

Page 11: **Supplementary Table S10**. Risk of outcome (1 day to 5 years) stratified by bone density and structure disorders

Page 12: **Supplementary Table S11**. Sensitivity analysis_exclusion of subjects with comorbidities related to other autoimmune diseases

Page 13: **Supplementary Table S12**. Sensitivity analysis_modified definition of the Sjögren cohort

Page 14: **Supplementary Table S13**. Sensitivity analysis_application of the same study design to different networks

**Supplementary Table S1. Sjögren syndrome-A extractable nuclear Ab or Sjögren syndrome-B extractable nuclear Ab codes used in the present study**

| Code | Descriptions |
|---|---|
| TNX:LG5158-3 | Sjögren syndrome-A extractable nuclear Ab [Units/volume] in Serum, Plasma or Blood |
| TNX:LG5161-7 | Sjögren syndrome-B extractable nuclear Ab [Units/volume] in Serum, Plasma or Blood |
| UMLS:LNC:8093-7 | Sjögren syndrome-A extractable nuclear Ab [Presence] in Serum |
| UMLS:LNC:31625-7 | Sjögren syndrome-A extractable nuclear IgG Ab [Presence] in Serum |
| UMLS:LNC:53016-2 | Sjögren syndrome-A extractable nuclear 52kD Ab [Presence] in Serum |
| UMLS:LNC:5351-2 | Sjögren syndrome-A extractable nuclear Ab [Presence] in Serum by Immunoassay |
| UMLS:LNC:17792-3 | Sjögren syndrome-A extractable nuclear Ab [Units/volume] in Serum |
| UMLS:LNC:33610-7 | Sjögren syndrome-A extractable nuclear IgG Ab [Units/volume] in Serum |
| UMLS:LNC:53019-6 | Sjögren syndrome-A extractable nuclear 60kD Ab [Units/volume] in Serum |
| UMLS:LNC:53017-0 | Sjögren syndrome-A extractable nuclear 52kD Ab [Units/volume] in Serum |
| UMLS:LNC:43100-7 | Sjögren syndrome-A and B extractable nuclear Ab panel - Serum |
| UMLS:LNC:33569-5 | Sjögren syndrome-A extractable nuclear Ab [Units/volume] in Serum by Immunoassay |
| UMLS:LNC:17585-1 | Sjögren syndrome-A extractable nuclear IgG Ab [Presence] in Serum by Immunoassay |
| UMLS:LNC:17584-4 | Sjögren syndrome-A extractable nuclear Ab [Units/volume] in Serum by Immunofluorescence |
| UMLS:LNC:29964-4 | Sjögren syndrome-A extractable nuclear IgG Ab [Units/volume] in Serum by Immunoassay |
| UMLS:LNC:56549-9 | Sjögren syndrome-A extractable nuclear 52kD Ab [Units/volume] in Serum by Immunoassay |
| UMLS:LNC:5352-0 | Sjögren syndrome-A extractable nuclear Ab [Presence] in Serum by Immune diffusion (ID) |
| UMLS:LNC:70257-1 | Sjögren syndrome-A extractable nuclear 52kD IgG Ab [Units/volume] in Serum by Immunoassay |
| UMLS:LNC:32041-6 | Sjögren syndrome-A extractable nuclear Ab+Sjögren syndrome-B extractable nuclear Ab [Units/volume] in Serum |
| UMLS:LNC:99139-8 | Sjögren syndrome-A extractable nuclear 52kD Ab [Units/volume] in Serum by Line blot |
| UMLS:LNC:8094-5 | Sjögren syndrome-B extractable nuclear Ab [Presence] in Serum |
| UMLS:LNC:17791-5 | Sjögren syndrome-B extractable nuclear Ab [Units/volume] in Serum |
| UMLS:LNC:31626-5 | Sjögren syndrome-B extractable nuclear IgG Ab [Presence] in Serum |
| UMLS:LNC:5353-8 | Sjögren syndrome-B extractable nuclear Ab [Presence] in Serum by Immunoassay |
| UMLS:LNC:33613-1 | Sjögren syndrome-B extractable nuclear IgG Ab [Units/volume] in Serum |
| UMLS:LNC:45142-7 | Sjögren syndrome-B extractable nuclear Ab [Units/volume] in Serum by Immunoassay |
| UMLS:LNC:17588-5 | Sjögren syndrome-B extractable nuclear IgG Ab [Presence] in Serum by Immunoassay |
| UMLS:LNC:33570-3 | Sjögren syndrome-B extractable nuclear Ab [Units/volume] in Serum by Immunoblot |
| UMLS:LNC:29965-1 | Sjögren syndrome-B extractable nuclear IgG Ab [Units/volume] in Serum by Immunoassay |
| UMLS:LNC:5354-6 | Sjögren syndrome-B extractable nuclear Ab [Presence] in Serum by Immune diffusion (ID) |
| UMLS:LNC:32041-6 | Sjögren syndrome-A extractable nuclear Ab+Sjögren syndrome-B extractable nuclear Ab [Units/volume] in Serum |
| TNX:LG5158-3 | Sjögren syndrome-A extractable nuclear Ab [Units/volume] in Serum, |

| | |
|---|---|
| | Plasma or Blood |
| TNX:LG5161-7 | Sjögren syndrome-B extractable nuclear Ab [Units/volume] in Serum, Plasma or Blood |
| UMLS:LNC:8093-7 | Sjögren syndrome-A extractable nuclear Ab [Presence] in Serum |
| UMLS:LNC:31625-7 | Sjögren syndrome-A extractable nuclear IgG Ab [Presence] in Serum |
| UMLS:LNC:53016-2 | Sjögren syndrome-A extractable nuclear 52kD Ab [Presence] in Serum |
| UMLS:LNC:5351-2 | Sjögren syndrome-A extractable nuclear Ab [Presence] in Serum by Immunoassay |
| UMLS:LNC:17792-3 | Sjögren syndrome-A extractable nuclear Ab [Units/volume] in Serum |
| UMLS:LNC:33610-7 | Sjögren syndrome-A extractable nuclear IgG Ab [Units/volume] in Serum |
| UMLS:LNC:53019-6 | Sjögren syndrome-A extractable nuclear 60kD Ab [Units/volume] in Serum |
| UMLS:LNC:53017-0 | Sjögren syndrome-A extractable nuclear 52kD Ab [Units/volume] in Serum |
| UMLS:LNC:43100-7 | Sjögren syndrome-A and B extractable nuclear Ab panel - Serum |
| UMLS:LNC:33569-5 | Sjögren syndrome-A extractable nuclear Ab [Units/volume] in Serum by Immunoassay |
| UMLS:LNC:17585-1 | Sjögren syndrome-A extractable nuclear IgG Ab [Presence] in Serum by Immunoassay |
| UMLS:LNC:17584-4 | Sjögren syndrome-A extractable nuclear Ab [Units/volume] in Serum by Immunofluorescence |
| UMLS:LNC:29964-4 | Sjögren syndrome-A extractable nuclear IgG Ab [Units/volume] in Serum by Immunoassay |
| UMLS:LNC:56549-9 | Sjögren syndrome-A extractable nuclear 52kD Ab [Units/volume] in Serum by Immunoassay |
| UMLS:LNC:5352-0 | Sjögren syndrome-A extractable nuclear Ab [Presence] in Serum by Immune diffusion (ID) |
| UMLS:LNC:70257-1 | Sjögren syndrome-A extractable nuclear 52kD IgG Ab [Units/volume] in Serum by Immunoassay |
| UMLS:LNC:32041-6 | Sjögren syndrome-A extractable nuclear Ab+Sjögren syndrome-B extractable nuclear Ab [Units/volume] in Serum |
| UMLS:LNC:99139-8 | Sjögren syndrome-A extractable nuclear 52kD Ab [Units/volume] in Serum by Line blot |
| UMLS:LNC:8094-5 | Sjögren syndrome-B extractable nuclear Ab [Presence] in Serum |
| UMLS:LNC:17791-5 | Sjögren syndrome-B extractable nuclear Ab [Units/volume] in Serum |
| UMLS:LNC:31626-5 | Sjögren syndrome-B extractable nuclear IgG Ab [Presence] in Serum |
| UMLS:LNC:5353-8 | Sjögren syndrome-B extractable nuclear Ab [Presence] in Serum by Immunoassay |
| UMLS:LNC:33613-1 | Sjögren syndrome-B extractable nuclear IgG Ab [Units/volume] in Serum |

Note:
UMLS, Unified Medical Language System
LNC, Logical Observation Identifiers Names and Codes (LOINC)
TNX, TriNetX

**Supplementary Table S2. Mastectomy or breast reconstruction procedure codes**

| Code | Descriptions |
|---|---|
| UMLS:ICD10CM:Z90.1 | Acquired absence of breast and nipple |
| UMLS:ICD9CM:85.4 | Mastectomy |
| UMLS:CPT:1015054 | Mastectomy Procedures |
| UMLS:SNOMED:428571003 | Mastectomy of left breast |
| UMLS:SNOMED:429400009 | Mastectomy of right breast |
| UMLS:CPT:19340 | Insertion of breast implant on same day of mastectomy (ie, immediate) |
| UMLS:CPT:19342 | Insertion or replacement of breast implant on separate day from mastectomy |
| UMLS:CPT:1036219 | Breast reconstruction |
| UMLS:SNOMED:61938004 | Breast reconstruction with free flap |
| UMLS:SNOMED:303445008 | Reconstruction of breast with flap |
| UMLS:HCPCS:S2066 | Breast reconstruction with gluteal artery perforator (gap) flap, including harvesting of the flap, microvascular transfer, closure of donor site and shaping the flap into a breast, unilateral |
| UMLS:HCPCS:S2068 | Breast reconstruction with deep inferior epigastric perforator (diep) flap or superficial inferior epigastric artery (siea) flap, including harvesting of the flap, microvascular transfer, closure of donor site and shaping the flap into a breast, unilateral |
| UMLS:HCPCS:S2067 | Breast reconstruction of a single breast with "stacked" deep inferior epigastric perforator (diep) flap(s) and/or gluteal artery perforator (gap) flap(s), including harvesting of the flap(s), microvascular transfer, closure of donor site(s) and shaping the flap into a breast, unilateral |

Note:
UMLS, Unified Medical Language System
ICD10CM, International Classification of Diseases, Tenth Revision, Clinical Modification
CPT, Current Procedural Terminology.
SNOMED, Systematized Nomenclature of Medicine.
HCPCS, Healthcare Common Procedure Coding System.

**Supplementary Table S3. Covariates of characteristics of study subjects**

| Baseline characteristics | Codes |
|---|---|
| **Demographic covariates** | |
| Potential health hazards related to housing and economic circumstances | ICD-10-CM: Z59 |
| Problems related to education and literacy | ICD-10-CM: Z55 |
| Family history of primary malignant neoplasm | ICD-10-CM: Z80 |
| Personal history of malignant neoplasm | ICD-10-CM: Z85 |
| **Lifestyle-related variables** | |
| Tobacco use | ICD-10-CM: Z72.0 |
| Nicotine dependence | ICD-10-CM: F17 |
| Alcohol related disorders | ICD-10-CM: F10 |
| **Medical utilization** | |
| Office or other outpatient services | CPT: 1013626 |
| Emergency department services | CPT: 1013711 |
| Hospital inpatient services | CPT: 1013659 |
| Preventive medicine services | CPT: 1013829 |
| **Comorbidities** | |
| Neoplasms | ICD-10-CM: C00-D49 |
| Benign breast disease | ICD-10-CM: D24 |
| Disorders of the blood and immune system | ICD-10-CM: D50-D89 |
| Hypertensive diseases | ICD-10-CM: I10-I1A |
| Cerebrovascular diseases | ICD-10-CM: I60-I69 |
| Atherosclerosis | ICD-10-CM: I70 |
| Diabetes mellitus | ICD-10-CM: E08-E13 |
| Vitamin D deficiency | ICD-10-CM: E55 |
| Overweight and obesity | ICD-10-CM: E66 |
| Hyperlipidemia | ICD-10-CM: E78.5 |
| Chronic lower respiratory diseases | ICD-10-CM: J40-J4A |
| Chronic lower respiratory diseases (CKD) | ICD-10-CM: N18 |
| Menopausal and female climacteric states | ICD-10-CM: N95.1 |
| Infertility | ICD-10-CM: N97 |
| Noninfective enteritis and colitis | ICD-10-CM: K50-K52 |
| Diseases of liver | ICD-10-CM: K70-K77 |
| Sleep disorders | ICD-10-CM: G47 |
| Depression | ICD-10-CM: F32 |
| Anxiety, and dissociative, stress-related, somatoform, and other nonpsychotic mental disorders | ICD-10-CM: F40-F48 |
| Rheumatoid arthritis with rheumatoid factor | ICD-10-CM: M05 |
| Other rheumatoid arthritis | ICD-10-CM: M06 |
| Systemic lupus erythematosus (SLE) | ICD-10-CM: M32 |
| Disorders of bone density and structure | ICD-10-CM: M80-M85 |
| **Procedures** | |
| Breast mammography | CPT: 1015090 |
| Radiation treatment management | CPT: 1010898 |
| Radiation treatment delivery | CPT: 1010875 |
| **Medication usage** | |
| Corticosteroids for systemic use | ATC: H02 |
| NSAIDs | ATC: M01A |
| Estrogens | ATC: G03C |
| Hormonal contraceptives for systemic use | ATC: G03A |
| Progestogens | ATC: G03D |
| Calcium | ATC: A12A |
| Vitamin D | ATC: A11CC |
| Bisphosphonates | ATC: M05BA |
| **Laboratory measurements** | |
| Sjogrens syndrome-A extractable nuclear Ab in Serum, Plasma or Blood | TNX: LG5158-3 |
| Sjogrens syndrome-B extractable nuclear Ab in Serum, Plasma or Blood | TNX: LG5161-7 |
| Testosterone in Serum, Plasma or Blood | TNX: LG11398-1 |

Note:
ICD-10-CM: International Classification of Diseases, Tenth Revision, Clinical Modification;
CPT: Current Procedural Terminology;
ATC: Anatomical Therapeutic Chemical Classification System;
NSAIDs: Anti-inflammatory and anti-rheumatic products, non-steroids;
TNX, TriNetX

**Supplementary Table S4. Risk of outcome-adjusted variables**

| Outcomes (Sjögren vs. Non- Sjögren cohort) | Hazard ratio (95% CI) | | | |
|---|---|---|---|---|
| | Model 1[a] (n=5104 vs. 2949492) | Model 2[b] (n=5104 vs. 5104) | Model 3[c] (n=5103 vs. 5103) | Model 4[d] (n=5102 vs. 5102) |
| **Breast cancer** | 1.160 (0.906-1.485) | 1.142 (0.804-1.621) | 1.079 (0.765-1.522) | 1.163 (0.821-1.647) |
| **Site** | | | | |
| Nipple and areola | 1.298 (0.487-3.461) | 0.611 (0.183-2.048) | 0.806 (0.225-2.886) | 1.234 (0.305-4.993) |
| Central portion | 1.372 (0.686-2.746) | 1.377 (0.498-3.808) | 2.663 (0.793-8.943) | 3.315 (0.874-12.56) |
| Upper-inner quadrant | 1.654 (0.916-2.990) | 1.409 (0.582-3.411) | 1.282 (0.543-3.029) | **3.256 (1.032-10.27)** |
| Lower-inner quadrant | 1.526 (0.635-3.671) | NA | 2.910 (0.562-15.06) | 1.052 (0.319-3.467) |
| Upper-outer quadrant | 0.926 (0.575-1.490) | 0.833 (0.446-1.555) | 0.789 (0.427-1.457) | 0.821 (0.444-1.521) |
| Lower-outer quadrant | 1.139 (0.511-2.538) | 1.803 (0.506-6.428) | 1.189 (0.382-3.702) | 1.501 (0.453-4.977) |
| Axillary tail of breast | NA | NA | NA | NA |
| Overlapping sites | **1.759 (1.147-2.700)** | 1.443 (0.760-2.741) | 1.914 (0.956-3.831) | 1.562 (0.820-2.974) |
| Unspecified site | 1.290 (0.999-1.665) | 1.378 (0.943-2.016) | 1.256 (0.869-1.816) | 1.247 (0.865-1.798) |
| **Receptor** | | | | |
| HER2 positive | NA | NA | NA | NA |
| Estrogen positive | 1.331 (0.976-1.815) | 1.200 (0.769-1.874) | 1.222 (0.782-1.907) | **1.640 (1.014-2.653)** |
| Progesterone positive | 0.600 (0.084-4.261) | 0.341 (0.036-3.284) | 0.531 (0.048-5.861) | 1.216 (0.075-19.65) |

Note: CI: Confidence interval. HER2: Human epidermal growth factor receptor 2. NA: Not available.
[a] Crude, before matching.
[b] Propensity score matching was performed on age and race.
[c] Propensity score matching was performed on age, race, social economic status, family history of malignant neoplasm, personal history of malignant neoplasm, lifestyles, breast mammography, and medical utilization.
[d] Propensity score matching was performed on age at index, race, social economic status, family history of malignant neoplasm, personal history of malignant neoplasm, lifestyles, breast mammography, medical utilization, comorbidities, and medicine usage.

**Supplementary Table S5. Risk of outcomes for different follow-up durations**

| Outcomes (Sjögren vs. Non- Sjögren cohort) | Adjusted hazard ratio (95% CI)[a] | | | |
|---|---|---|---|---|
| | 1 day to 180 days | 1 day to 1y | 1 day to 3y | 1 day to 5y |
| **Breast cancer** | 1.636 (0.877-3.049) | 1.596 (0.966-2.637) | 1.111 (0.774-1.595) | 1.079 (0.765-1.522) |
| **Site** | | | | |
| Nipple and areola | 1.008 (0.142-7.157) | 1.523 (0.255-9.117) | 0.649 (0.155-2.721) | 0.806 (0.225-2.886) |
| Central portion | NA | NA | 2.679 (0.519-13.81) | 2.663 (0.793-8.943) |
| Upper-inner quadrant | 3.020 (0.610-14.96) | 3.538 (0.735-17.03) | 1.372 (0.541-3.478) | 1.282 (0.543-3.029) |
| Lower-inner quadrant | NA | NA | 2.230 (0.407-12.21) | 2.910 (0.562-15.06) |
| Upper-outer quadrant | 7.027 (0.865-57.11) | 2.052 (0.770-5.469) | 0.838 (0.440-1.598) | 0.789 (0.427-1.457) |
| Lower-outer quadrant | 3.032 (0.315-29.15) | 4.095 (0.458-36.64) | 1.378 (0.420-4.528) | 1.189 (0.382-3.702) |
| Axillary tail of breast | NA | NA | NA | NA |
| Overlapping sites | 6.041 (0.727-50.17) | **3.761 (1.049-13.48)** | 1.812 (0.879-3.738) | 1.914 (0.956-3.831) |
| Unspecified site | 1.923 (0.927-3.989) | **1.850 (1.071-3.197)** | 1.258 (0.852-1.857) | 1.256 (0.869-1.816) |
| **Receptor** | | | | |
| HER2 positive | NA | NA | NA | NA |
| Estrogen receptor positive | **3.627 (1.347-9.769)** | **2.765 (1.338-5.712)** | 1.324 (0.832-2.106) | 1.222 (0.782-1.907) |
| Progesterone receptor positive | NA | 1.021 (0.064-16.33) | 0.531 (0.048-5.861) | 0.531 (0.048-5.861) |

Note: CI: Confidence interval. HER2: Human epidermal growth factor receptor 2. NA: Not available.
[a] Propensity score matching was performed on age, race, social economic status, family history of malignant neoplasm, personal history of malignant neoplasm, lifestyles, breast mammography, and medical utilization.

**Supplementary Table S6. Risk of outcome (1 day to 5 years) stratified by age**

| Outcomes (Sjögren cohort vs. Non- Sjögren cohort) | Adjusted hazard ratio (95% CI)[a] | |
|---|---|---|
| | 30-64 years (n=3599 vs. 3599) | ≧ 65 years (n=1695 vs. 1695) |
| **Breast cancer** | 0.954 (0.582-1.564) | 1.003 (0.651-1.545) |
| **Site** | | |
| Nipple and areola | 1.157 (0.233-5.757) | 2.137 (0.193-23.60) |
| Central portion | 0.697 (0.126-3.857) | 3.494 (0.703-17.37) |
| Upper-inner quadrant | 0.993 (0.300-3.281) | 1.762 (0.495-6.270) |
| Lower-inner quadrant | 0.376 (0.039-3.622) | 2.767 (0.535-14.30) |
| Upper-outer quadrant | 1.024 (0.416-2.524) | 1.373 (0.526-3.582) |
| Lower-outer quadrant | 0.508 (0.046-5.607) | 2.012 (0.478-8.468) |
| Axillary tail of breast | NA | NA |
| Overlapping sites | 1.552 (0.607-3.963) | 3.097 (0.984-9.748) |
| Unspecified site | 0.954 (0.573-1.591) | 1.035 (0.657-1.630) |
| **Receptor** | | |
| HER2 positive | NA | NA |
| Estrogen receptor positive | 0.968 (0.506-1.853) | 1.746 (0.945-3.226) |
| Progesterone receptor positive | NA | NA |

Note: CI: Confidence interval. HER2: Human epidermal growth factor receptor 2. NA: Not available.

[a] Propensity score matching was performed on age, race, social economic status, family history of malignant neoplasm, personal history of malignant neoplasm, lifestyles, breast mammography, and medical utilization.

**Supplementary Table S7. Risk of outcome (1 day to 5 years) stratified by race**

| Outcomes<br>(Sjögren cohort vs. Non-Sjögren cohort) | Adjusted hazard ratio (95% CI)[a] | | |
|---|---|---|---|
| | White<br>(n=3380 vs. 3380) | Black or African American<br>(n=794 vs. 794) | Asian<br>(n=240 vs. 240) |
| **Breast cancer** | 1.474 (0.945-2.302) | 0.956 (0.321-2.851) | 1.551 (0.413-5.819) |
| **Site** | | | |
| Nipple and areola | 0.645 (0.057-7.256) | 1.054 (0.066-16.86) | 1.562 (0.098-24.96) |
| Central portion | 1.943 (0.544-6.934) | NA | NA |
| Upper-inner quadrant | 1.591 (0.550-4.602) | NA | 1.039 (0.065-16.62) |
| Lower-inner quadrant | 3.219 (0.617-16.79) | NA | NA |
| Upper-outer quadrant | 1.281 (0.543-3.026) | 0.735 (0.122-4.419) | 0.612 (0.055-6.853) |
| Lower-outer quadrant | 2.995 (0.578-15.52) | NA | NA |
| Axillary tail of breast | NA | NA | NA |
| Overlapping sites | **3.343 (1.315-8.498)** | 0.615 (0.055-6.825) | 2.748 (0.247-30.53) |
| Unspecified site | 1.348 (0.855-2.124) | 0.963 (0.323-2.874) | 1.580 (0.422-5.921) |
| **Receptor** | | | |
| HER2 positive | NA | NA | NA |
| Estrogen receptor positive | **1.860 (1.031-3.353)** | 0.744 (0.124-4.468) | 1.235 (0.247-6.181) |
| Progesterone receptor positive | NA | NA | NA |

Note: CI: Confidence interval. HER2: Human epidermal growth factor receptor 2. NA: Not available.

[a] Propensity score matching was performed on age, race, social economic status, family history of malignant neoplasm, personal history of malignant neoplasm, lifestyles, breast mammography, and medical utilization.

**Supplementary Table S8. Risk of outcome (1 day to 5 years) stratified by obesity**

| Outcomes<br>(Sjögren cohort vs. Non- Sjögren cohort) | Adjusted hazard ratio (95% CI)[a] | |
|---|---|---|
| | Obese[b]<br>(n=927 vs. 927) | Non-obese[c]<br>(n=2964 vs. 2964) |
| **Breast cancer** | 1.249 (0.507-3.077) | 1.354 (0.845-2.172) |
| **Site** | | |
| Nipple and areola | NA | 1.827 (0.303-11.02) |
| Central portion | 1.320 (0.082-21.30) | 1.700 (0.536-5.393) |
| Upper-inner quadrant | 1.754 (0.292-10.55) | 2.967 (0.567-15.52) |
| Lower-inner quadrant | NA | 1.268 (0.249-6.446) |
| Upper-outer quadrant | 0.621 (0.056-6.844) | 1.445 (0.621-3.361) |
| Lower-outer quadrant | NA | 1.501 (0.334-6.734) |
| Axillary tail of breast | NA | NA |
| Overlapping sites | 0.758 (0.127-4.544) | **4.034 (1.309-12.42)** |
| Unspecified site | 1.265 (0.488-3.284) | 1.503 (0.912-2.478) |
| **Receptor** | | |
| HER2 positive | NA | NA |
| Estrogen receptor positive | 1.424 (0.382-5.309) | 1.495 (0.816-2.740) |
| Progesterone receptor positive | NA | NA |

Note: CI: Confidence interval. HER2: Human epidermal growth factor receptor 2. NA: Not available.

[a] Propensity score matching was performed on age, race, social economic status, family history of malignant neoplasm, personal history of malignant neoplasm, lifestyles, breast mammography, and medical utilization.

[b] Diagnosed overweight and obesity (ICD10CM:E66) or BMI at least 30 kg/m$^2$ within 1 year before the index date.

[c] Never diagnosed overweight and obesity (ICD10CM:E66) or BMI at least 30 kg/m$^2$ documented in their electric medical records.

**Supplementary Table S9. Risk of outcome (1 day to 5 years) stratified by DM**

| Outcomes<br>(Sjögren cohort vs. Non- Sjögren cohort) | Adjusted hazard ratio (95% CI)[a] | |
|---|---|---|
| | With DM[b]<br>(n=382 vs. 382) | Without DM[c]<br>(n=4163 vs. 4163) |
| **Breast cancer** | 2.732 (0.704-10.59) | 1.178 (0.791-1.753) |
| **Site** | | |
| Nipple and areola | NA | 0.572 (0.104-3.136) |
| Central portion | NA | 0.593 (0.205-1.712) |
| Upper-inner quadrant | 3.544 (0.366-34.30) | 1.822 (0.593-5.594) |
| Lower-inner quadrant | NA | 1.668 (0.369-7.538) |
| Upper-outer quadrant | NA | 1.078 (0.557-2.083) |
| Lower-outer quadrant | NA | 0.976 (0.296-3.218) |
| Axillary tail of breast | NA | NA |
| Overlapping sites | 1.213 (0.076-19.46) | 1.462 (0.734-2.911) |
| Unspecified site | 2.350 (0.586-9.432) | 1.178 (0.781-1.776) |
| **Receptor** | | |
| HER2 positive | NA | NA |
| Estrogen receptor positive | 1.177 (0.237-5.858) | 1.635 (0.954-2.801) |
| Progesterone receptor positive | NA | NA |

Note: CI: Confidence interval. DM: Diabetes mellitus. HER2: Human epidermal growth factor receptor 2. NA: Not available.
[a] Propensity score matching was performed on age, race, social economic status, family history of malignant neoplasm, personal history of malignant neoplasm, lifestyles, breast mammography, and medical utilization.
[b] Diagnosed DM (ICD10CM:E08-E13) within 1 year before the index date.
[c] Never diagnosed DM (ICD10CM:E08-E13) documented in their electric medical records.

**Supplementary Table S10. Risk of outcome (1 day to 5 years) stratified by bone density and structure disorders**

| Outcomes<br>(Sjögren cohort vs. Non- Sjögren cohort) | Adjusted hazard ratio (95% CI)[a] | |
|---|---|---|
| | With<br>bone density and structure disorders[b]<br>(n=381 vs. 381) | Without<br>bone density and structure disorders[c]<br>(n=3346 vs. 3346) |
| **Breast cancer** | 0.681 (0.267-1.739) | 1.217 (0.648-2.287) |
| **Site** | | |
| Nipple and areola | NA | NA |
| Central portion | 2.318 (0.210-25.63) | 1.280 (0.179-9.142) |
| Upper-inner quadrant | NA | 4.435 (0.492-39.93) |
| Lower-inner quadrant | NA | 1.561 (0.098-24.99) |
| Upper-outer quadrant | 0.340 (0.070-1.650) | 0.995 (0.302-3.279) |
| Lower-outer quadrant | NA | NA |
| Axillary tail of breast | NA | NA |
| Overlapping sites | 1.096 (0.154-7.783) | NA |
| Unspecified site | 0.781 (0.297-2.053) | 1.474 (0.747-2.908) |
| **Receptor** | | |
| HER2 positive | NA | NA |
| Estrogen receptor positive | 0.584 (0.174-1.957) | 1.120 (0.443-2.832) |
| Progesterone receptor positive | NA | NA |

Note: CI: Confidence interval. HER2: Human epidermal growth factor receptor 2. NA: Not available.
[a] Propensity score matching was performed on age, race, social economic status, family history of malignant neoplasm, personal history of malignant neoplasm, lifestyles, breast mammography, and medical utilization.
[b] Diagnosed bone density and structure disorders (ICD10CM:M80-M85) within 1 year before the index date.
[c] Never diagnosed bone density and structure disorders (ICD10CM:M80-M85) documented in their electric medical records.

**Supplementary Table S11. Sensitivity analysis_exclusion of subjects with comorbidities related to other autoimmune diseases**

| Outcomes | Patients with outcome | | Adjusted hazard ratio (95% CI)[a] |
|---|---|---|---|
| | Sjögren cohort (n=3357) | Non-Sjögren cohort (n=3357) | |
| **Breast cancer** | 45 | 50 | 1.059 (0.706-1.588) |
| **Site** | | | |
| Nipple and areola | 10 | 10 | 0.897 (0.200-4.027) |
| Central portion | 10 | 10 | 0.907 (0.286-2.876) |
| Upper-inner quadrant | 10 | 10 | 3.930 (0.809-19.07) |
| Lower-inner quadrant | 10 | 10 | 0.847 (0.267-2.691) |
| Upper-outer quadrant | 12 | 16 | 0.877 (0.414-1.860) |
| Lower-outer quadrant | 10 | 10 | 0.732 (0.238-2.246) |
| Axillary tail of breast | 0 | 10 | NA |
| Overlapping sites | 16 | 16 | 1.170 (0.584-2.345) |
| Unspecified site | 42 | 41 | 1.211 (0.785-1.866) |
| **Receptor** | | | |
| HER2 positive | 0 | 0 | NA |
| Estrogen receptor positive | 30 | 30 | 1.134 (0.682-1.884) |
| Progesterone receptor positive | 10 | 10 | 0.522 (0.047-5.762) |

Note: CI: Confidence interval. HER2: Human epidermal growth factor receptor 2. NA: Not available.
If the patient is less or equal to 10, results show the count as 10.
[a] Propensity score matching was performed on age, race, social economic status, family history of malignant neoplasm, personal history of malignant neoplasm, lifestyles, breast mammography, and medical utilization.

**Supplementary Table S12. Sensitivity analysis_modified definition of the Sjögren cohort**

| Outcomes | Patients with outcome | | Adjusted hazard ratio (95% CI)[a] |
|---|---|---|---|
| | Sjögren cohort (n=27117) | Non-Sjögren cohort (n=27117) | |
| **Breast cancer** | 477 | 444 | 0.955 (0.840-1.087) |
| **Site** | | | |
| Nipple and areola | 22 | 29 | 0.671 (0.385-1.167) |
| Central portion | 45 | 60 | **0.655 (0.445-0.964)** |
| Upper-inner quadrant | 60 | 59 | 0.899 (0.627-1.287) |
| Lower-inner quadrant | 37 | 31 | 1.050 (0.651-1.692) |
| Upper-outer quadrant | 158 | 132 | 1.060 (0.841-1.336) |
| Lower-outer quadrant | 45 | 54 | 0.732 (0.493-1.087) |
| Axillary tail of breast | 10 | 10 | 0.775 (0.281-2.137) |
| Overlapping sites | 122 | 89 | 1.207 (0.919-1.587) |
| Unspecified site | 406 | 386 | 0.934 (0.812-1.073) |
| **Receptor** | | | |
| HER2 positive | 0 | 10 | NA |
| Estrogen receptor positive | 271 | 256 | 0.939 (0.792-1.114) |
| Progesterone receptor positive | 14 | 18 | 0.696 (0.346-1.399) |

Note: CI: Confidence interval. HER2: Human epidermal growth factor receptor 2. NA: Not available.
If the patient is less or equal to 10, results show the count as 10.
[a] Propensity score matching was performed on age, race, social economic status, family history of malignant neoplasm, personal history of malignant neoplasm, lifestyles, breast mammography, and medical utilization.

**Supplementary Table S13. Sensitivity analysis_application of the same study design to different networks**

| Outcomes (Sjögren cohort vs. Non- Sjögren cohort) | Adjusted hazard ratio (95% CI)[a] | | |
|---|---|---|---|
| | Global network (n=8238 vs. 8238) | US network (n=5103 vs. 5103) | APAC network (n=886 vs. 886) |
| **Breast cancer** | 1.025 (0.770-1.364)* | 1.079 (0.765-1.522) | 0.742 (0.254-2.172) |
| **Site** | | | |
| Nipple and areola | 1.821 (0.533-6.224) | 0.806 (0.225-2.886) | NA |
| Central portion | 0.986 (0.450-2.162) | 2.663 (0.793-8.943) | NA |
| Upper-inner quadrant | 0.795 (0.376-1.681) | 1.282 (0.543-3.029) | NA |
| Lower-inner quadrant | 1.586 (0.447-5.624) | 2.910 (0.562-15.06) | NA |
| Upper-outer quadrant | 0.795 (0.478-1.321) | 0.789 (0.427-1.457) | 0.485 (0.093-2.523) |
| Lower-outer quadrant | 0.968 (0.373-2.513) | 1.189 (0.382-3.702) | NA |
| Axillary tail of breast | NA | NA | NA |
| Overlapping sites | 1.130 (0.659-1.938) | 1.914 (0.956-3.831) | 1.391 (0.232-8.335) |
| Unspecified site | 1.014 (0.742-1.386)* | 1.256 (0.869-1.816) | 0.708 (0.139-3.597) |
| **Receptor** | | | |
| HER2 positive | NA | NA | NA |
| Estrogen receptor positive | 0.893 (0.587-1.360)* | 1.222 (0.782-1.907) | NA |
| Progesterone receptor positive | 0.211 (0.025-1.809) | 0.531 (0.048-5.861) | NA |

Note: CI: Confidence interval. HER2: Human epidermal growth factor receptor 2. NA: Not available.
[a] Propensity score matching was performed on age, race, social economic status, family history of malignant neoplasm, personal history of malignant neoplasm, lifestyles, breast mammography, and medical utilization.
* Proportionality <0.001.
